# Exercise Interventions in Polycystic Ovary Syndrome: A Systematic Review and Meta-Analysis

**DOI:** 10.3389/fphys.2020.00606

**Published:** 2020-07-07

**Authors:** Rhiannon K. Patten, Russell A. Boyle, Trine Moholdt, Ida Kiel, William G. Hopkins, Cheryce L. Harrison, Nigel K. Stepto

**Affiliations:** ^1^Institute for Health and Sport, Victoria University, Melbourne, VIC, Australia; ^2^Department of Circulation and Medical Imaging, Norwegian University of Science and Technology, Trondheim, Norway; ^3^Monash Centre for Health Research and Implementation (MCHRI), School of Public Health and Preventive Medicine, Monash University, Melbourne, VIC, Australia; ^4^Australian Institute for Musculoskeletal Science (AIMSS), Victoria University, Melbourne, VIC, Australia

**Keywords:** lifestyle intervention, physical activity, resistance training, exercise intensity, cardiorespiratory fitness, metabolic health, insulin resistance, high-intensity interval training

## Abstract

**Background:** Polycystic ovary syndrome (PCOS) is a common and complex endocrinopathy with reproductive and metabolic manifestations. Exercise training has consistently been found to result in improved clinical outcomes in women with PCOS, but shortfalls with exercise prescription are evident. The aim of this systematic review and meta-analysis was to identify exercise intervention characteristics that provide favourable outcomes in women with PCOS.

**Methods:** A systematic review of published literature was conducted using EBSCOhost and Ovid Medline up to May 2019. The review adheres to the Preferred Reporting Items for Systematic Review and Meta-Analyses (PRISMA) guidelines as per our PROSPERO protocol (CRD42018088367). Randomised controlled trials, non-randomised controlled trials, and uncontrolled trials that evaluated an exercise intervention of at least moderate intensity in women with PCOS were included. Meta-analyses were performed using general linear mixed modelling and Bayesian inferences about effect magnitudes.

**Results:** Thirty-three articles were identified for systematic review of which 19 were meta-analysed. Intervention duration ranged from 6 to 26 weeks. A total number of 777 women were included in the meta-analysis. The meta-analysis found that improvements in health outcomes are more dependent on exercise intensity rather than dose. Fixed effects analysis reported a moderate increase in VO_2peak_ (24.2%; 90% CL, 18.5–30.1), and small reductions in HOMA-IR (−36.2%; 90% CL, −55.3 to −9.0), and waist circumference (−4.2%; 90% CL −6.0 to −2.3) as a result of vigorous intensity exercise. These results are confirmed in the predicted analysis which reported the greatest improvements in VO_2peak_, BMI, and waist circumference after vigorous intensity exercise alone or when combined with diet, particularly for women with clinically adverse baseline values.

**Conclusions:** Exercise training in the management of PCOS is becoming more common. Results from our analysis support the use of exercise and suggest that vigorous intensity exercise may have the greatest impact on cardiorespiratory fitness, body composition, and insulin resistance. Our results indicate that, a minimum of 120 min of vigorous intensity per week is needed to provide favourable health outcomes for women with PCOS with studies of longer duration required to evaluate outcomes with sustained exercise.

## Introduction

Polycystic ovary syndrome (PCOS) is one of the most common endocrine conditions, affecting 8–13% of reproductive aged women (Teede et al., 2018a). PCOS is complex with diverse features including reproductive, metabolic, and mental health complications. PCOS is diagnosed via the internationally endorsed Rotterdam criteria, which require the presence of two or more features including clinical or biochemical signs of hyperandrogenism, oligo- or anovulation, and polycystic ovaries on ultrasound, with the exclusion of other aetiologies (Rotterdam EA-SPCWG, 2004). Although not currently recognised in the diagnostic criteria, insulin resistance is a key aetiological factor contributing to the severity of reproductive and metabolic features (Poretsky et al., 1999; Altuntas et al., 2003), with obesity known to exacerbate the severity of clinical symptoms (Lim et al., 2012). Consequently, women with PCOS are at a two to eight times greater risk of developing impaired glucose tolerance and type 2 diabetes mellitus compared to women without PCOS (Moran et al., 2010).

Exercise is well-established as a therapy for preventing and managing chronic diseases in the general population (Booth et al., 2000; Roberts and Barnard, 2005), and in women with PCOS (Wild et al., 2010; Teede et al., 2018a; Stepto et al., 2019). The beneficial effects of exercise in women with PCOS have been summarised in several recent systematic reviews and meta-analyses (Benham et al., 2018; Kite et al., 2019). In addition, the international evidence-based guidelines for the management of PCOS recommend lifestyle intervention, including exercise training and diet, as the first line of therapy to improve general health, hormonal outcomes, and quality of life (Teede et al., 2018a). However, the studies utilised in the development of the guidelines were limited to a small number of randomised controlled trials (RCTs), resulting in a general consensus recommendation of exercise rather than a clear exercise prescription for the management of PCOS (Teede et al., 2018b; Stepto et al., 2019). In particular, there is uncertainty about suitable intensity, duration, and modality of exercise, and the interaction between exercise and diet. We have addressed this uncertainty by meta-analysing the effects of exercise characteristics on key clinical markers in women with PCOS, with the aim of assisting clinicians with exercise prescription and guiding future research in women with PCOS.

## Methods

### Protocol and Registration

This systematic review and meta-analysis was conducted and reported in accordance with the Preferred Reporting Items for Systematic Reviews and Meta-Analyses (PRISMA) and was registered on the International prospective register for systematic reviews (PROSPERO) CRD42018088367.

### Search Strategy, Study Selection, and Data Extraction

We performed a systematic search of the literature to May 2019 inclusive using EBSCOhost (MEDLINE, SPORTDiscus, PsycINFO, CINAHL) and Ovid Medline. The search was limited to peer reviewed, published, English language articles from 1980-current. The search terms were modified when required for each database and are reported in Supplementary Table 1. The reference lists of other review articles were searched to identify other potential eligible studies. After removal of duplicates, two reviewers (RB and RP) independently screened articles by title and abstract. Subsequently, the same reviewers independently completed full-text screening. Any discrepancies were resolved by consensus or by consultation with a third reviewer (NS). After full-text screening, data extraction of eligible studies was performed independently by RB and RP using a pre-determined extraction form.

Where required, authors were contacted via email using an institutional email address in order to obtain additional or raw data. Following a second email, if no response was received within 14 days, the article was excluded from the meta-analysis. Where multiple publications resulted from the same trial, results were combined, and only one result (largest participant number) for each outcome was used in the analysis.

### Eligibility Criteria

The Participant, Intervention, Comparison, Outcomes, and Studies (PICOS) framework was used for this systematic review (Table 1). Briefly, included studies involved women aged 18–45 (pre-menopausal) and with a diagnosis of PCOS via any established diagnostic criteria. The interventions included RCTs, non-randomised controlled trials, and uncontrolled trials that had a pre-post design and reported outcomes of an exercise training intervention greater than two weeks in duration, and of moderate intensity or greater. Exercise intensity was categorised according to Norton et al. (2010), classified as moderate (55 to <70% HR_max_ or 40 to < 60% VO_2max_), vigorous (70 to <90% HR_max_ or 60 to <85% VO_2max_), or high (≥90% HR_max_ or ≥85% VO_2max_) intensity. Two weeks was used as a minimum intervention duration in order to capture the effects of exercise training. We accepted exercise interventions that included aerobic exercise, resistance training, or a combination. Exercise interventions that were combined with a drug therapy that may affect the outcomes measures were excluded. Exercise interventions that were combined with a dietary intervention or dietary advice were included. Comparison groups consisted of a no exercise control group or a diet only group. The primary outcomes specified for the meta-analysis were peak oxygen consumption (VO_2peak_) used to measure cardiorespiratory fitness, homeostatic model assessment of insulin resistance (HOMA-IR) to measure insulin resistance, free androgen index (FAI) to measure androgens, and body mass index (BMI), and waist circumference to assess weight related outcomes. Secondary outcomes were those that were included in the systematic review only and consist of additional reproductive, cardio-metabolic, or anthropometric outcomes (Table 1).

**Table 1:** Eligibility criteria for study inclusion.

**Participant**	**Intervention**	**Comparator**	**Outcome**	**Study design**	**Limits**
**Inclusion criteria**					
Diagnosed with PCOS using any established definition Premenopausal women aged 18–45. Any weight category	Any exercise intervention that is: Supervised or unsupervised Greater than 2 weeks in duration Moderate intensity (>55% HR_max_ or >40% VO_2peak_) or above	No exercise control group Diet only group	Cardiorespiratory Fitness—VO_2peak_ Metabolic—measures of insulin sensitivity, lipids Body composition—weight, BMI, W/H ratio, waist circumference Reproductive—menstrual regularities, hormonal markers	RCT Clinical trial Non-RCT Pilot Feasibility Parallel	English language Human trials Peer reviewed

*HR_max_, Maximal Heart Rate; VO_2peak_, Peak Oxygen Consumption; BMI, Body Mass Index; W/H Ratio, Waist to Hip Ratio; RCT, Randomised Controlled Trial*.

### Assessment of Risk of Bias in Included Studies

The Downs and Black Checklist for the Assessment of Methodological Quality (Downs and Black, 1998) was used to evaluate the included randomised and non-randomised studies. Two reviewers (RB and RP) independently assessed the methodological quality and disagreements were resolved by consensus. Questions regarding blinding of participants were removed from the checklist (Supplementary Table 2). As per our previous meta-analysis (Cassar et al., 2016) publication bias was assessed by examining a scatter plot of *t*-statistic associated with each study estimate value contributing to the study-estimate random effect versus log of the standard error of the effect. No outliers or publication bias was identified using this approach.

### Data Analysis

The meta-analyses were performed with the general linear mixed-model procedure in the Statistical Analysis System (Version 9.4, SAS Institute, Cary, NC, USA). The fixed effects in the model were used to estimate the main effects of exercise and its modifiers. A nominal variable represented type of exercise (moderate, vigorous, resistance, control) and a linear numeric variable represented total dose of any exercise (in hours). A nominal variable with two levels (exercise, control) was interacted with the baseline value of the dependant variable and with a dummy variable representing a dietary co-intervention (described as either a structured dietary plan, dietary advice, or guidance) to estimate respectively the modifying effects of baseline and of diet in exercise and control groups. The linear numeric effect of baseline HOMA-IR produced unrealistic predictions at low values of this dependant variable, so the numeric variable was replaced by a nominal variable with three levels defined by the following: low, <2.1; moderate, 2.1–3.4; high, >3.4.

A random effect representing the identity of each study estimate was included to allow for differences in the means of study estimates not accounted for by the fixed effects, and an additional random effect representing estimate identity accounted for study clusters of estimates (control and/or one or more experimental estimates). In the mixed-model, each study estimate was weighted by the inverse of the square of its standard error (*SE*), and the random effects were estimated by setting the residual variance to unity (Yang, 2003). The standard error of each estimate was either derived as the standard deviation (SD) of change scores divided by the square root of the sample size or was computed with a spreadsheet from either the exact *p*-value or compatibility intervals for the mean change. For estimates where the standard error could not be derived from the data, it was imputed from the error of measurement averaged over other similar estimates (Supplementary Table 3). The number of imputed standard errors represented 0.1–3% of the total number, depending on the meta-analysed measure.

All study-estimates, standard errors, and baseline between-subject *SD*s were converted to factor effects by dividing by the group mean and then log-transformed for the meta-analysis. The qualitative magnitudes of meta-analysed mean effects were evaluated via standardisation using magnitude thresholds provided by an appropriate baseline between-subject *SD* (Hopkins et al., 2009). This *SD* was derived by combining (via variances) the mean of the study *SD*s with the between-study *SD* of the study baseline means, to represent the *SD* of women drawn randomly from a population of sub-populations. The threshold for the smallest important effect (0.2 *SD*) was rounded down towards the mean of the study *SD* to allow for differences in study means to be due partly to differences in assay technique; the thresholds for small, moderate, large, very large, and extremely large were 1, 3, 6, 10, and 20 times the smallest important threshold, respectively (corresponding to 0.2, 0.6, 1.2, 2.0, and 4.0 times the *SD*, Supplementary Table 4) (Cassar et al., 2016). These thresholds were also used to evaluate the magnitude of the numeric linear modifying effects of baseline and training duration, by multiplying the beta-coefficients (slopes) in the model by two between-subject *SD* and between-study *SD*, respectively (Hopkins et al., 2009). The thresholds were halved for evaluation of the magnitude of the random-effect *SD*s (Smith and Hopkins, 2011). The meta-analysis also provided predicted population and individual-setting effects of various combinations of exercise, diet and baseline values for the dependent variable. The predicted effects for individual settings had the same value as the predicted population mean effects, but their compatibility intervals were wider, owing to the contribution of the between- and within-study random effects.

Uncertainty in the estimates of effects is presented as 90% compatibility limits. Probabilistic decisions about true (large-sample) magnitudes accounting for the uncertainty were based on one-sided hypothesis tests of substantial magnitudes (Lakens et al., 2018). The *p*-value for rejecting a hypothesis of a given magnitude was the area of the sampling t distribution of the effect statistic with values of that magnitude. For effect modifiers, random-effect *SD*s, and predicted population mean effects, hypotheses of substantial decrease, and increase were rejected if their respective *p*-values were <0.05. For predicted effects in individual settings, hypotheses of harm, and benefit were rejected if the respective *p*-values were <0.005 and 0.25. If one hypothesis was rejected, the *p*-value for the other hypothesis was interpreted as evidence *for* that hypothesis, since the *p*-value corresponds to the posterior probability of the magnitude of the true effect in a reference Bayesian analysis with a minimally informative prior (Hopkins and Batterham, 2018; Hopkins, 2019). The *p*-value is reported qualitatively using the following scale: 0.25–0.75, possibly; 0.75–0.95, likely; 0.95–0.995, very likely; >0.995, most likely (Hopkins et al., 2009). This scale was also used to interpret the posterior probability of a true trivial effect, which is given by the area of the sampling distribution in trivial values. If neither hypothesis was rejected, the magnitude of the effect was considered to be unclear, and the magnitude of the effect is shown without a probabilistic qualifier. To reduce inflation of error arising from the large number of effects investigated, effects were considered decisive with more conservative *p*-value thresholds (*p* < 0.01 for a substantial decrease or increase; *p* < 0.001 for harm; *p* < 0.05 for benefit) and are formatted bold in tables and figures.

## Results

**Figure 1:**
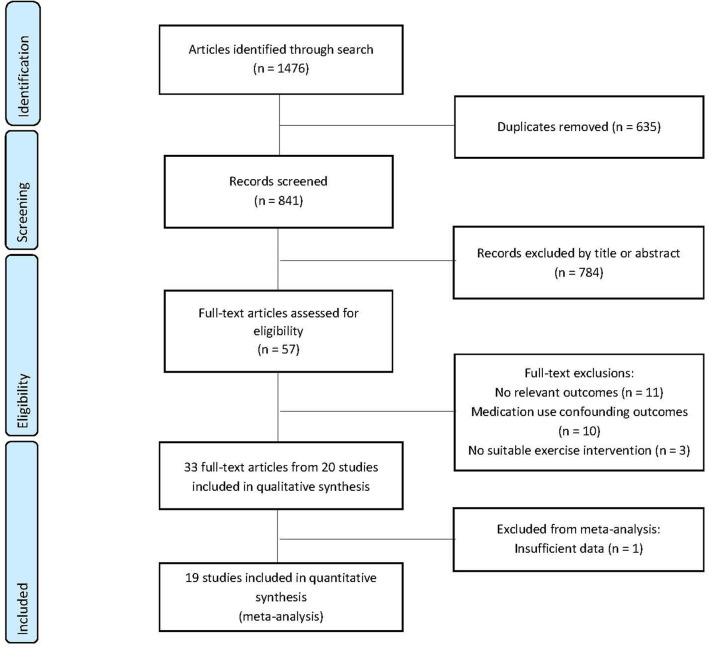
Preferred Reporting Items for Systematic Reviews and Meta-Analyses (PRISMA) study selection flow diagram.

The combined searches identified 1,476 papers for review. Of these, 635 were excluded due to duplication, 784 were removed by title and abstract screening, with 57 full-text articles reviewed. Twenty-four of these were excluded due to medication use, lack of data on outcomes of interest and intervention type, with 33 publications deemed suitable for inclusion in the systematic review. Multiple publications were identified for six exercise intervention studies and thereafter amalgamated with the largest reported number (*N*) for outcome measures of interest carried forward for meta-analysis. The remaining 20 articles were included in the systematic review with one article excluded from the meta-analysis due to using a non-parametric analysis (Brown et al., 2009) (Figure 1). A summary of study and participant characteristics, exercise intervention, and study outcomes is reported in Table 2 and results of the methodological quality are presented in **Table 4** (for full results see Supplementary Table 5).

**Table 2:** Summary of studies identified for systematic review detailing participants, intervention characteristics and main outcomes measures.

**Study**	**Study design**	**QA score**	**Exercise *N* (total *N*)**	**Participant characteristics**	**PCOS diagnostic criteria**	**Exercise intervention characteristics**	**CRF outcomes**	**Cardiometabolic outcomes**	**Hormonal and reproductive outcomes**	**Body composition outcomes**
**Included in meta-analysis**										
Almenning et al. (2015)	RCT	17	HIIT = 8 RT = 8 (25)	Age = 27.2 ± 5.5 RT: BMI = 27.4 ± 6.9 HIIT: BMI = 26.1 ± 6.5	Rotterdam	Type: Aerobic Intervals or RT Frequency: 3/weeks Intensity: Vigorous-High (70–95% HR_max_) Duration: 10 weeks Supervision: Partial	HIIT: ↑ VO2peak RT: No change	HIIT: ↓ HOMA-IR, ↓ Fasting Insulin, ↑ HDL RT: No change	HIIT: No change RT: ↓ AMH ↑ SHBG, ↓ FAI	HIIT: ↓ Fat mass, ↓ BF% RT: ↓ BF%, ↑ Fat free mass
Bruner et al. (2006)	RCT	10	7 (12)	Age = 32.3 ± 1 BMI = 36.2 ± 2	Rotterdam	Type: Aerobic and RT Frequency: 3/weeks Intensity: Vigorous (70–85% HR_max_) Duration: 12 weeks Supervision: Full	↑ VO_2peak_	↓ Fasting Insulin	No change	↓ WC
Costa et al. (2018)	RCT	17	14 (27)	Age = 27.6 ± 4.5 BMI = 25-39.9	Rotterdam	Type: Aerobic Frequency: 3/weeks Intensity: Moderate-Vigorous (60–85% HR_max_) Duration: 16 weeks Supervision: Full	↑ VO_2peak_	No change	Not measured	↓ BMI, ↓ WC
Curi et al. (2012)	Randomised parallel trial	11	12 (27)	Age = 26.3 ± 1.4 BMI = 31.8 ± 1.6	Rotterdam	Type: Aerobic Frequency: N/R Intensity: Moderate Duration: 26 weeks Supervision: None	Not measured	No change	No change	↓ BMI, ↓ WC
Giallauria et al. (2008)	Uncontrolled trial	17	62 (124)	Age = 22.8 ± 3.7 BMI = 29.2 ± 2.9	Rotterdam	Type: Aerobic Frequency: 3/weeks Intensity: Vigorous (60–70% VO_2peak_) Duration: 12 weeks Supervision: Full	↑ VO_2peak_	↓ Fasting Insulin, ↓ AUCi_ns_, ↑ AUC_glu_/AUC_ins_	↑ SHBG	↓ BMI, ↓ W/H
Hutchison et al. (2011) Moran et al. (2011) Hutchison et al. (2012) Harrison et al. (2012)	Uncontrolled trial	15 13 13 14	13 (21)	Age = 29.75 ± 1.4 BMI = 35.6 ± 5.8	NIH	Type: Aerobic Intervals Frequency: 3/weeks Intensity: Vigorous (75–100% HR_max_) Duration: 12 weeks Supervision: Full	↑ VO_2peak_	↑ GIR, ↓ Fasting Insulin, ↓ HOMA-IR.	↓ AMH No other changes	↓ BMI
Ladson et al. (2011)	RCT	17	59 (114)	Age = 28.8 ± 4.6 BMI = 38.3 ± 8	NIH	Type: Aerobic Frequency: ≥2/weeks Intensity: Moderate Duration: 26 weeks Supervision: Partial	No change	↑ AUC_glu_, ↑ HDL	No change	↓ WC
Miranda-Furtado et al. (2016) Kogure et al. (2016) Kogure et al. (2018)	Uncontrolled trial	13 15 14	45 (97)	Age = 28.1 ± 5.4 BMI = 28.4 ± 6	Rotterdam	Type: RT Frequency: 3/weeks Intensity: % of 1RM Duration: 16 weeks Supervision: Full	Not measured	No change	↓ T, ↓ FAI, ↑ SHBG	↓ WC
Moro et al. (2009) Redman et al. (2011) Covington et al. (2015) Covington et al. (2016)	Uncontrolled trial	10 10 9 9	8 (16)	Age = 25.6 ± 3.1 BMI = 32.1 ± 5.2	Rotterdam	Type: Aerobic Frequency: 5/weeks Intensity: Moderate (55% VO_2peak_) Duration: 16 weeks Supervision: Full	↑ VO_2peak_	↑ GDR, ↑ HDL	No change	No change
Nybacka et al. (2011) Nybacka et al. (2013)	RCT	10 12	Exercise = 17, Diet and exercise = 12 (43)	Age = 31.8 ± 4.9 BMI = 34.9 ± 5.3	Rotterdam	Type: Aerobic Frequency: 2–3/weeks Intensity: Moderate Duration: 16 weeks Supervision: Full	Not measured	No change	↑ Menstrual Cyclicity. No change in hormonal markers.	↓ BMI
Orio et al. (2016)	RCT	18	39 (136)	Age = 25.9 ± 2.7 BMI = 26.7 ± 2.8	NIH	Type: Aerobic Frequency: 3/weeks Intensity: Vigorous (60–70% VO_2peak_) Duration: 24 weeks Supervision: Full	↑ VO2_2peak_	↓ Fasting Insulin, ↓ HOMA-IR, ↑ GIR, ↓ AUC_ins_, ↓ Total Cholesterol, ↑ HDL, ↓ LDL	No change	↓ BMI, ↓ W/H
Orio et al. (2008)	Non-randomised parallel study	14	32 (64)	Age = 18-40 BMI = 28.9 ± 3	Rotterdam	Type: Aerobic Frequency: 3/weeks Intensity: Vigorous (60–70% VO_2peak_) Duration: 24 weeks Supervision: Full	↑ VO_2peak_	↓ Fasting Insulin, ↓ AUCins, ↑ AUCglu/ins, ↑ HDL, ↓ LDL	No change	↓ BMI ↓ WC, ↓ W/H
Randeva et al. (2002)	Uncontrolled trail	12	12 (21)	Age = 29.7 ± 6.8 BMI = 33.9 ± 4.5	Rotterdam	Type: Aerobic Frequency: 3/weeks Intensity: Moderate Duration: 26 weeks Supervision: None	↑ VO_2peak_	No Change	Not measured	↓ W/H
Roessler et al. (2013)	Randomised crossover trial	15	8 (17)	Age = 31 (SEM−3) BMI = 34.8 (SEM-2.5)	Rotterdam	Type: Aerobic and aerobic intervals Frequency: 3/weeks Intensity: Vigorous-High (70–75 and 80–100% HR_max_) Duration: 8 weeks Supervision: N/R	↑ VO_2peak_	Not measured	Not measured	↓ Weight, ↓ BMI, ↓ WC
Sprung et al. (2013a)	Single-arm trial	15	6 (12)	Age = 28 (25 – 31) BMI = 31 (28 – 34)	Rotterdam	Type: Aerobic Frequency: 3–5/weeks Intensity: Moderate (30–60% HRR) Duration: 16 weeks Supervision: Full	↑ VO_2peak_	No change	No change	No change
Sprung et al. (2013b)	Non-RCT	14	10 (17)	Age = 29 ± 7 BMI = 34 ± 6	Rotterdam	Type: Aerobic Frequency: 3–5/weeks Intensity: Moderate (30–60% HRR) Duration: 16 weeks Supervision: Full	↑ VO_2peak_	↓ Total Cholesterol, ↓ LDL No change in insulin sensitivity	No change	No change
Jedel et al. (2011) Stener-Victorin et al. (2009) Stener-Victorin et al. (2012)	RCT	16 13 16	30 (74)	Age = 30.2 ± 4.7 BMI = 27.7 ± 6.4	Rotterdam	Type: Aerobic Frequency: 3/weeks Intensity: Moderate Duration: 16 weeks Supervision: None	↑ VO_2peak_	No change	↑ SHBG, ↓ Free T, ↓ Estradiol	↓ Weight, ↓ BMI
Thomson et al. (2008) Thomson et al. (2012) Thomson et al. (2016)	RCT	11 13 10	Diet and aerobic exercise = 18, Diet and combined exercise = 20 (52)	Age = 29.3 ± 6.8 BMI = 36.1 ± 4.8	Rotterdam	Type: Aerobic, RT or combined aerobic and RT Frequency: 5/weeks Intensity: Moderate-Vigorous (60–80% HR_max_ and 50–75% of 1RM) Duration: 20 weeks Supervision: N/R	↑ VO_2peak_	↓ HOMA-IR, ↓ Fasting Glucose, ↓ Fasting Insulin, ↓ Lipids	↓ T, ↓ FAI, ↑ SHBG, ↑ Menstrual Cyclicity	↓ Weight, ↓ WC, ↓ Fat mass, ↓ BF%
Vigorito et al. (2007)	RCT	15	45 (90)	Age = 21.7 ± 2.3 BMI = 29.3 ± 2.9	Rotterdam	Type: Aerobic Frequency: 3/weeks Intensity: Vigorous (60-70% VO_2peak_) Duration: 12 weeks Supervision: Full	↑ VO_2peak_	↓ Fasting Insulin, ↓ AUCins, ↑ AUCglu/AUCins	↑ Menstrual Cyclicity. No change in hormonal markers.	↓ WC, ↓ BMI, ↓ W/H
**Included for systematic review only**										
Brown et al. (2009)	RCT	17	8 (20)	Age = 36.5 (5) BMI = 37.9 (9.4)	NIH	Type: Aerobic Frequency: 3–5/weeks Intensity: Moderate (40–60% VO_2peak_) Duration: 20–24 weeks Supervision: Full	↑ VO_2peak_	No change	Not measured	No change

*QA, Quality Assessment; CRF- Cardiorespiratory Fitness; RCT, Randomised Controlled Trial; NIH, National Institute of Health; HIIT, High Intensity Interval Training; RT, Resistance Training; BMI, Body Mass Index; HR_max_, Maximal Heart Rate; VO_2peak_, Peak Oxygen Uptake; HRR, Heart Rate Reserve; N/R, Not Reported; ↑ Increase, ↓ Decrease, HOMA-IR, Homeostatic Model Assessment of Insulin Resistance; AUC, Area Under the Curve; GDR, Glucose Disposal Rate; GIR, Glucose Infusion Rate; HDL, High Density Lipoproteins; LDL, Low Density Lipoproteins; AMH, Anti-Mullerian Hormone; SHBG, Sex Hormone Binding Globulin; FAI, Free Androgen Index; T, Testosterone; Free T, Free Testosterone; BF%, Body Fat Percentage; WC, Waist Circumference; W/H, Waist to Hip Ratio*.

### Summary of Articles

Of the 20 trials included in the systematic review, 10 were RCTs (Bruner et al., 2006; Vigorito et al., 2007; Thomson et al., 2008; Brown et al., 2009; Jedel et al., 2011; Ladson et al., 2011; Nybacka et al., 2011; Almenning et al., 2015; Orio et al., 2016; Costa et al., 2018), five non-randomised uncontrolled trials (Randeva et al., 2002; Giallauria et al., 2008; Hutchison et al., 2012; Covington et al., 2016; Miranda-Furtado et al., 2016), one randomised parallel trial (Curi et al., 2012), and one non-randomised parallel trial (Orio et al., 2008), one randomised cross-over trial (Roessler et al., 2013), one single arm trial (Sprung et al., 2013a), and one non-randomised controlled trial (Sprung et al., 2013b). The mean age of participants ranged from 22 to 32 years. Baseline BMI ranged from 26.1 to 38.3 kg/m^2^. Participants from 16 studies were diagnosed according to the Rotterdam criteria (Randeva et al., 2002; Bruner et al., 2006; Vigorito et al., 2007; Giallauria et al., 2008; Orio et al., 2008; Thomson et al., 2008; Jedel et al., 2011; Nybacka et al., 2011; Curi et al., 2012; Roessler et al., 2013; Sprung et al., 2013a,b; Almenning et al., 2015; Covington et al., 2016; Miranda-Furtado et al., 2016; Costa et al., 2018) and the remaining four studies used the NIH diagnostic criteria (Brown et al., 2009; Ladson et al., 2011; Hutchison et al., 2012; Orio et al., 2016). Sample sizes ranged from 5 to 62. Of the 20 reported interventions, 13 had full intervention supervision (Bruner et al., 2006; Vigorito et al., 2007; Giallauria et al., 2008; Orio et al., 2008, 2016; Brown et al., 2009; Nybacka et al., 2011; Hutchison et al., 2012; Sprung et al., 2013a,b; Covington et al., 2016; Costa et al., 2018), two had partial supervision (at least one supervised session per week; Ladson et al., 2011; Almenning et al., 2015), three had no supervision (Randeva et al., 2002; Jedel et al., 2011; Curi et al., 2012), and two did not report on supervision (Thomson et al., 2008; Roessler et al., 2013). Practitioners that supervised the exercise intervention included exercise physiologists, physiotherapists and physical activity educators.

Fourteen studies involved only a continuous aerobic intervention (Randeva et al., 2002; Vigorito et al., 2007; Giallauria et al., 2008; Orio et al., 2008, 2016; Brown et al., 2009; Jedel et al., 2011; Ladson et al., 2011; Nybacka et al., 2011; Curi et al., 2012; Sprung et al., 2013a,b; Covington et al., 2016; Costa et al., 2018), three had a high intensity aerobic interval training group (Hutchison et al., 2012; Roessler et al., 2013; Almenning et al., 2015), three had a resistance training group (Thomson et al., 2008; Almenning et al., 2015; Miranda-Furtado et al., 2016) and two had a combined resistance training and aerobic group (Bruner et al., 2006; Thomson et al., 2008). Of the interventions that included an aerobic exercise component, three included high intensity aerobic intervals (Hutchison et al., 2012; Roessler et al., 2013; Almenning et al., 2015), five included vigorous intensity exercise (Bruner et al., 2006; Vigorito et al., 2007; Giallauria et al., 2008; Orio et al., 2008, 2016), two included moderate to vigorous intensity exercise (Thomson et al., 2008; Costa et al., 2018) and nine included moderate intensity exercise (Randeva et al., 2002; Brown et al., 2009; Jedel et al., 2011; Ladson et al., 2011; Nybacka et al., 2011; Curi et al., 2012; Sprung et al., 2013a,b; Covington et al., 2016). The duration and frequency of exercise interventions ranged from 8 to 26 weeks and 2 to 5 sessions per week, respectively. The length of individual session duration varied, ranging from 30 to 90 min.

Seventeen studies measured VO_2peak_ (Randeva et al., 2002; Bruner et al., 2006; Vigorito et al., 2007; Giallauria et al., 2008; Orio et al., 2008, 2016; Thomson et al., 2008; Brown et al., 2009; Jedel et al., 2011; Ladson et al., 2011; Hutchison et al., 2012; Roessler et al., 2013; Sprung et al., 2013a,b; Almenning et al., 2015; Covington et al., 2016; Costa et al., 2018). Of these studies, 16 reported significant improvements following an exercise intervention (Randeva et al., 2002; Bruner et al., 2006; Vigorito et al., 2007; Giallauria et al., 2008; Orio et al., 2008, 2016; Thomson et al., 2008; Brown et al., 2009; Jedel et al., 2011; Hutchison et al., 2012; Roessler et al., 2013; Sprung et al., 2013a,b; Almenning et al., 2015; Covington et al., 2016; Costa et al., 2018). Nineteen studies measured metabolic outcomes (Randeva et al., 2002; Bruner et al., 2006; Vigorito et al., 2007; Giallauria et al., 2008; Orio et al., 2008, 2016; Thomson et al., 2008; Brown et al., 2009; Jedel et al., 2011; Ladson et al., 2011; Nybacka et al., 2011; Curi et al., 2012; Hutchison et al., 2012; Sprung et al., 2013a,b; Almenning et al., 2015; Covington et al., 2016; Miranda-Furtado et al., 2016; Costa et al., 2018), 11 of which reported significant changes in at least one marker of metabolic health (Bruner et al., 2006; Vigorito et al., 2007; Giallauria et al., 2008; Orio et al., 2008, 2016; Thomson et al., 2008; Ladson et al., 2011; Hutchison et al., 2012; Sprung et al., 2013b; Almenning et al., 2015; Covington et al., 2016). Four studies reported significant decreases in HOMA-IR (Thomson et al., 2008; Hutchison et al., 2012; Almenning et al., 2015; Orio et al., 2016), eight studies reported decreases in fasting insulin levels (Bruner et al., 2006; Vigorito et al., 2007; Giallauria et al., 2008; Orio et al., 2008, 2016; Thomson et al., 2008; Hutchison et al., 2012; Almenning et al., 2015), three reported significant improvements in glucose infusion or glucose disposal rates (Hutchison et al., 2012; Covington et al., 2016; Orio et al., 2016), five studies reported positive increases in HDL (Orio et al., 2008, 2016; Ladson et al., 2011; Almenning et al., 2015; Covington et al., 2016) and eight studies reported no changes in markers of metabolic health following an exercise intervention (Randeva et al., 2002; Brown et al., 2009; Jedel et al., 2011; Nybacka et al., 2011; Curi et al., 2012; Sprung et al., 2013a; Miranda-Furtado et al., 2016; Costa et al., 2018). Sixteen studies measured changes in hormonal markers and reproductive health (Randeva et al., 2002; Bruner et al., 2006; Vigorito et al., 2007; Giallauria et al., 2008; Orio et al., 2008, 2016; Thomson et al., 2008; Jedel et al., 2011; Ladson et al., 2011; Nybacka et al., 2011; Curi et al., 2012; Hutchison et al., 2012; Sprung et al., 2013a,b; Almenning et al., 2015; Covington et al., 2016; Miranda-Furtado et al., 2016). Five studies reported a significant increase in sex hormone binding globulin (SHBG) levels (Giallauria et al., 2008; Thomson et al., 2008; Jedel et al., 2011; Almenning et al., 2015; Miranda-Furtado et al., 2016), three studies reported significant decreases in FAI (Thomson et al., 2008; Almenning et al., 2015; Miranda-Furtado et al., 2016), two studies reported significant decreases in anti-mullerian hormone (AMH) levels (Hutchison et al., 2012; Almenning et al., 2015), three studies reported improvements in menstrual cyclicity (Vigorito et al., 2007; Thomson et al., 2008; Nybacka et al., 2011) and eight studies reported no changes in reproductive outcomes post exercise intervention (Bruner et al., 2006; Orio et al., 2008, 2016; Ladson et al., 2011; Curi et al., 2012; Sprung et al., 2013a,b; Covington et al., 2016). All 20 studies measured changes in body composition following exercise intervention. Sixteen reported significant changes in at least one measure of body composition (Randeva et al., 2002; Bruner et al., 2006; Vigorito et al., 2007; Giallauria et al., 2008; Orio et al., 2008, 2016; Thomson et al., 2008; Jedel et al., 2011; Ladson et al., 2011; Nybacka et al., 2011; Curi et al., 2012; Hutchison et al., 2012; Roessler et al., 2013; Almenning et al., 2015; Miranda-Furtado et al., 2016; Costa et al., 2018). Ten studies reported significant decreases in BMI (Vigorito et al., 2007; Giallauria et al., 2008; Orio et al., 2008, 2016; Jedel et al., 2011; Nybacka et al., 2011; Curi et al., 2012; Hutchison et al., 2012; Roessler et al., 2013; Costa et al., 2018), nine reported decreases in waist circumference (Bruner et al., 2006; Vigorito et al., 2007; Thomson et al., 2008; Ladson et al., 2011; Curi et al., 2012; Roessler et al., 2013; Miranda-Furtado et al., 2016; Orio et al., 2016; Costa et al., 2018), five reported decreases in waist to hip ratio (Randeva et al., 2002; Vigorito et al., 2007; Giallauria et al., 2008; Orio et al., 2008, 2016), three report decreased weight (Thomson et al., 2008; Jedel et al., 2011; Roessler et al., 2013). The remaining four studies reported no significant changes in any measure of body composition (Brown et al., 2009; Sprung et al., 2013a,b; Covington et al., 2016).

### Meta-Analysis

**Figure 2:**
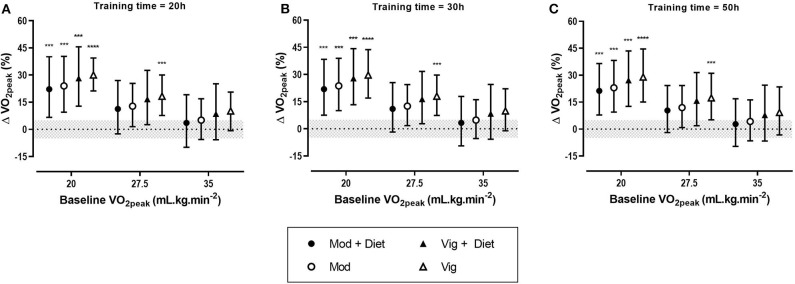
Predicted effects of exercise alone or exercise plus diet versus a control group on peak oxygen uptake (VO_2peak_) after 20 h **(A)**, 30 h **(B)**, and 50 h **(C)** of moderate (Mod), or vigorous (Vig) intensity exercise in an individual study setting. Clear effects are shown with the probability of a true substantial change (***very likely, ****most likely). Magnitudes in bold are clear with 99% compatibility intervals.

**Figure 3:**
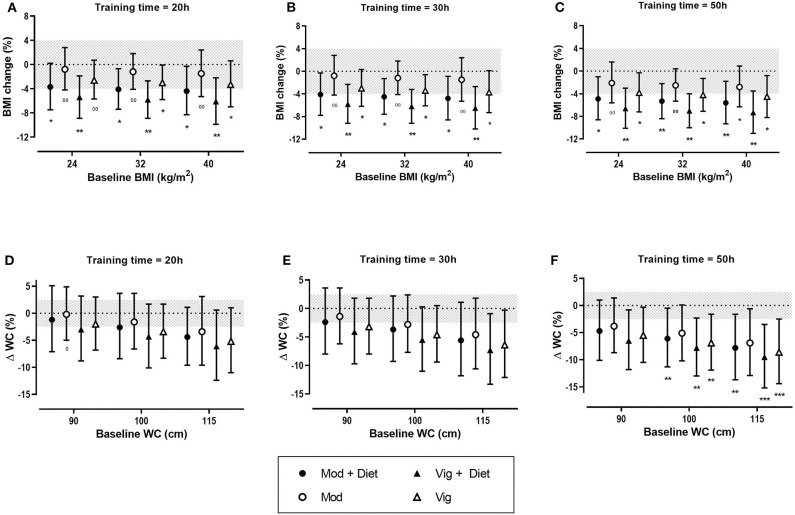
Predicted effects of exercise alone or exercise plus diet versus a control group on body mass index (BMI) and waist circumference (WC) after 20 h **(A,D)**, 30 h **(B,E)**, and 50 h **(C,F)** of moderate (Mod), or vigorous (Vig) intensity exercise in an individual study setting. Clear effects are shown with the probability of either a true substantial change (*possibly, **likely, ***very likely) and/or a true trivial change (^0^possibly, ^00^likely). Magnitudes in bold are clear with 99% compatibility intervals.

**Figure 4:**
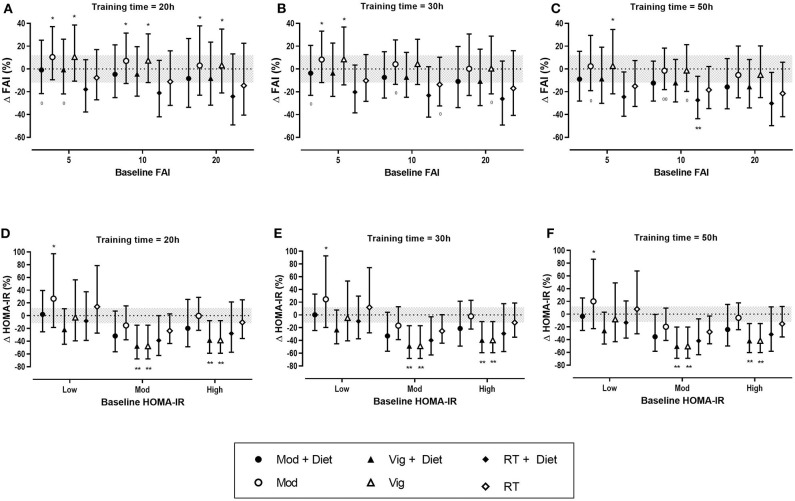
Predicted effects of exercise alone or exercise plus diet versus a control group on free androgen index (FAI) and homeostatic model assessment of insulin resistance (HOMA-IR) after 20 h **(A,D)**, 30 h **(B,E)**, and 50 h **(C,F)** of moderate (Mod), vigorous (Vig) intensity exercise, or resistance training (RT) in an individual study setting. Clear effects are shown with the probability of either a true substantial change (*possibly, **likely) and/or a true trivial change (^0^possibly, ^00^likely). Baseline HOMA-IR: Low, <2.1%; Moderate (Mod), 2.1–3.4%; High, >3.4%. Magnitudes in bold are clear with 99% compatibility intervals.

The results from the meta-analysis of the effect of exercise characteristics on cardiorespiratory fitness measured by VO_2peak_, body composition (BMI and WC), insulin resistance (HOMA-IR) and hyperandrogenism as measured by FAI are presented as the population mean effects and modifying effects of exercise characteristics (Tables 2, 3) and predicted effects of exercise across various durations and baseline values (Figures 2–**4**).

**Table 3:** Meta-analysed effects on peak oxygen uptake (VO_2peak_), body mass index (BMI) and waist circumference expressed as population mean effects in control and exercise groups, and as modifying effects of exercise duration, baseline, and dietary co-intervention.

	**$\text{V}{\text{O}}_{2\text{peak}}^{\text{b}}$**			**BMI^c^**			**Waist circumference^d^**		
	**Mean (%)**	**90% CL (%)**	**Magnitude**	**Mean (%)**	**90% CL (%)**	**Magnitude**	**Mean (%)**	**90% CL (%)**	**Magnitude**
**Population mean effects^a^**									
Control group	1.0	−2.3, 4.4	**Trivial^ooo^**	0.7	−0.2, 1.7	**Trivial^0000^**	0.8	−1.2, 2.8	Trivial^00^
Moderate exercise	18.4	11.2, 26.1	Moderate↑^****^	−0.9	−2.0, 0.3	**Trivial^0000^**	−1.6	−3.7, 0.5	**Trivial^00^**
Vigorous exercise	24.2	18.5, 30.1	Moderate↑^****^	−2.6	−3.6, −1.7	**Trivial^000^**	−3.4	−5.3, −1.5	Small↓^**^
Moderate—control group	17.2	9.7, 25.3	Moderate↑^***^	−1.6	−2.9, −0.2	**Trivial^0000^**	−2.4	−4.1, −0.6	Small↓^*^
Vigorous—control group	22.9	16.9, 29.2	Moderate↑^****^	−3.3	−4.5, −2.2	**Trivial^00^**	−4.2	−6.0, −2.3	Small↓^**^
**Modifying effects**									
Baseline in control group	4.9	−1.4, 11.6	Trivial^0^	1.3	−1.2, 3.9	**Trivial^000^**	0.8	−2.9, 4.7	Trivial
Baseline in exercise group	−10.3	−17.6, −2.4	Small↓^**^	0.8	−1.3, 3.0	**Trivial^000^**	−2.2	−5.9, 1.8	Small↓^*^
30 h of exercise duration	−0.8	−7.8, 6.8	Trivial	−1.3	−2.8, 0.2	**Trivial^0000^**	−3.6	−6.0, −1.2	Small↓^**^
Diet in control group	−1.9	−11.1, 8.2	Trivial	−5.6	−8.4, −2.7	Small↓^**^	−7.5	−10.7, −4.3	Moderate↓^***^
Diet in exercise group	−1.4	−10.0, 8.0	Trivial	−2.9	−4.6, −1.2	**Trivial^00^**	−1.0	−4.3, 2.5	Trivial^00^

a *Evaluated at mean baseline (VO_2peak_ = 24 mL.kg.min^-1^, BMI = 31 kg.m^2^, waist circumference = 97 cm), training time = 30 h, and no dietary co-intervention*.

b *Modifying effect of baseline is evaluated per 70% difference in baseline value*.

c *Modifying effect of baseline is evaluated per 40% difference in baseline value*.

d *Modifying effect of baseline is evaluated per 25% difference in baseline value*.

*90% CL 90% compatibility limits, ↑ increase, ↓ decrease*.

Effects are shown with their observed magnitudes, determined by standardisation. Clear effects are show with the probability of either a true substantial change (^*^possibly, ^**^likely, ^***^very likely, ^****^most likely) and/or a true trivial change (^0^possibly, ^00^likely, ^000^very likely, ^0000^most likely). Magnitudes in bold are clear with 99% compatibility intervals.

#### Effect of Exercise on VO_2peak_

Meta-analysis from 16 studies with a total population of 600 women with PCOS, revealed moderate improvements in VO_2peak_ after moderate and vigorous intensity aerobic exercise, with the largest increase was seen after vigorous intensity exercise (Table 3). Across all conditions, the modifying effects of intervention duration and dietary co-intervention on VO_2peak_ were trivial.

The predicted effects analysis showed that irrespective of training dose, vigorous intensity aerobic exercise alone had the most substantial increase in VO_2peak_ (Figure 2). Moreover, it is clear that baseline value plays a major role in the magnitude of improvements, with lower baseline VO_2peak_ values resulting in the largest improvements.

#### Effects of Exercise on BMI

Meta-analysis of 17 studies which included a total of 759 women with PCOS were included to determine the effect of exercise on BMI. The predicted mean results of each intervention were trivial (Table 3). The largest reductions in BMI were reported for women undertaking vigorous intensity exercise compared to a control group. The modifying effects of baseline BMI, duration and diet were also trivial with the exception of the effect of diet in a control group which resulted in a small decrease in BMI.

In the predicted effects analysis, training dose appears to have a limited effect on BMI outcome. The addition of diet intervention to exercise resulted in clear reductions in BMI. Notably, vigorous intensity exercise combined with a dietary intervention potentiated BMI changes, with small to moderate reductions of BMI across all baseline BMIs and training durations (Figure 3).

#### Effects of Exercise on Waist Circumference (WC)

Thirteen studies which included 463 women overall were used in this analysis of the fixed effects of exercise on WC. Vigorous intensity exercise when compared to a control group resulted in the greatest reductions in WC. The modifying effect of diet in a control group resulted in a moderate decrease in WC. In contrast, there was a trivial effect of diet in the exercise group (Table 3).

The predicted effects analysis found the greatest improvement in WC with a combined vigorous intensity aerobic exercise and diet across the range of baseline WCs (Figure 3). Greater improvements were seen in women with a higher baseline WC. It was also apparent that training dose had a clear moderating effect on WC with greater decreases being reported after 50 h of exercise in comparison to 20 h of exercise (Figure 3).

#### Effects of Exercise on Free Androgen Index (FAI)

Sixteen studies were included in the meta-analysis of exercise-induced changes in hyperandrogenism as measured by FAI and included a total of 667 women with PCOS. Of the 16 studies, three included a resistance training intervention. Our analysis showed that the greatest improvements in FAI occurred after resistance training (Table 3). Both moderate and vigorous aerobic exercise resulted in only trivial changes. The effect of diet resulted in a small decrease in FAI in both the exercise and control groups (Table 3).

The predicted effects analysis also reported trivial changes in FAI after aerobic exercise. Resistance training when combined with diet had the largest effect on FAI, resulting in small to moderate reductions of FAI across all baseline values and training doses, however the results were mostly unclear (Figure 4). It is apparent from the analysis that training duration plays a role in the extent of improvements in FAI, with the largest effects being seen after 50 h of exercise.

#### Effects of Exercise on Insulin Resistance (HOMA-IR)

Eleven studies (307 women with PCOS) were included in the meta-analysis for the effect of exercise on HOMA-IR. Vigorous intensity aerobic exercise and resistance training both resulted in moderate reductions in HOMA-IR when compared to a control group (Table 4). The modifying effect of diet on HOMA-IR resulted in a moderate reduction in a no-exercise control group, and a small reduction in an exercise group. The modifying effect of baseline in a control group resulted in moderate increases in HOMA-IR but only trivial effects in an exercise group (Table 4).

**Table 4:** Meta-analysed effects on homeostatic model assessment of insulin resistance (HOMA-IR) and free androgen index (FAI) expressed as population mean effects in control and exercise groups, and as modifying effects of exercise duration, baseline, and dietary co-intervention.

	**HOMA-IR^b^**			**FAI^c^**		
	**Mean (%)**	**90% CL (%)**	**Magnitude**	**Mean (%)**	**90% CL (%)**	**Magnitude**
**Population mean effects^a^**						
Control group	32.4	1.3, 72.9	Small↑^**^	−2.9	−11.1, 6.1	**Trivial^000^**
Moderate exercise	10.1	−6.7, 30.0	Small↑^*^	2.2	−6.7, 12.0	Trivial^000^
Vigorous exercise	−15.6	−33.2, 6.7	Small↓^*^	2.4	−7.4, 13.2	Trvial^00^
Resistance exercise	−1.0	−14.4, 14.5	Trivial	−15.3	−28.4, 0.2	Small↓^*^
Moderate—control group	−16.8	−38.8, 13.1	Small↓^*^	5.2	−6.2, 18.0	Trivial^00^
Vigorous—control group	−36.2	−55.3, −9.0	Moderate↓^**^	5.4	−6.1, 18.4	Trivial^00^
Resistance—control group	−25.2	−44.4, 0.6	Moderate↓^**^	−12.3	−27.3, 4.6	Small↓^*^
**Modifying effects**						
Baseline in control group	43.9	11.7, 85.4	Moderate↑^**^	7.5	−11.5, 30.6	Small↑^*^
Baseline in exercise group	13.1	−25.1, 70.9	Trivial	1.1	−15.1, 20.3	Trivial
30 h of exercise duration	−5.4	−35.6, 38.9	Trivial	−8.1	−20.3, 6.0	Small↓^*^
Diet in control group	−43.1	−58.9, −21.3	Small↓^***^	−21.9	−32.8, −9.2	Small↓^**^
Diet in exercise group	−19.5	−44.5, 16.6	Trivial	−11.1	−20.6, −0.4	Small↓^*^

a *Evaluated at mean baseline (HOMA-IR=moderate, FAI = 8.4%), training time = 30 h, and no dietary co-intervention*.

b *Modifying effect of baseline is evaluated for high versus low baseline*.

c *Modifying effect of baseline is evaluated for a 3.0-fold difference in baseline*.

*90% CL 90% compatibility limits, ↑ increase, ↓ decrease*.

Effects are shown with their observed magnitudes, determined by standardisation. Clear effects are show with the probability of either a true substantial change (^*^possibly, ^**^likely, ^***^very likely) and/or a true trivial change (^00^likely, ^000^very likely). Magnitudes in bold are clear with 99% compatibility intervals.

The predicted effects analysis on the effects of exercise on HOMA-IR show that clear improvements in HOMA-IR were only seen after vigorous intensity exercise both alone and vigorous exercise when combined with a dietary intervention, resulting in moderate reductions, irrespective of training dose (Figure 4).

## Discussion

This is the first systematic review and meta-analysis to evaluate the effectiveness of varying exercise intensities and the moderating effects of dietary co-intervention, training dose, and baseline values on cardiorespiratory, metabolic, and reproductive health outcomes in women with PCOS. Results from this systematic review demonstrate clear improvements in several of these outcomes following an exercise intervention in women with PCOS. The most consistent improvements were seen with cardiorespiratory fitness (VO_2peak_), BMI, WC, and various markers of metabolic health, including fasting insulin, and HOMA-IR. These results are supported by our meta-analysis, which revealed improvements in VO_2peak_, body composition and insulin sensitivity following an exercise intervention, particularly when compared to a no-exercise control group. Vigorous intensity exercise, both alone and when combined with a dietary intervention, resulted in the greatest improvements in health parameters in both the fixed effects and predicted effects analyses. Moderate intensity exercise resulted in clear improvements in VO_2peak_, WC, and BMI when combined with diet as seen in the predicted analysis. Interestingly, resistance training showed promising improvements in FAI and HOMA-IR in both fixed effect and predicted analyses, however further research is required to confirm these improvements.

This systematic evaluation of exercise interventions align with identified knowledge gaps in current international evidence-based guidelines (Teede et al., 2018a; Stepto et al., 2019) and meta-analyses (Benham et al., 2018; Kite et al., 2019) recently undertaken in this population of women. There is substantial evidence that supports the effectiveness of aerobic exercise training for improving some health outcomes in women with PCOS. In particular, aerobic exercise of various intensities has consistently been found to result in improvements in VO_2peak_ in women with PCOS (Haqq et al., 2015; Kite et al., 2019; Stepto et al., 2019). VO_2peak_ is a measure of cardiorespiratory fitness and is an important indicator of health and mortality (Blair et al., 1989). Individuals with a lower VO_2peak_ are at an increased risk of all-cause mortality and morbidity with the risk of death being more dependent on cardiorespiratory fitness than BMI (Barry et al., 2014). To illustrate this point, with each 3.5 mL/kg/min increase in VO_2peak_, there is an associated 13% risk reduction from all-cause mortality (Kodama et al., 2009). Based on observed improvements from our meta-analysis, women with PCOS and relatively low VO_2peak_ of 24mL/kg/min are likely to experience a ~30% risk reduction in all-cause mortality after 30 h of vigorous intensity exercise over 10–12 weeks, irrespective of any dietary co-intervention. An increase in exercise intensity becomes of paramount importance for improving VO_2peak_ in women with high baseline values. These results expand on existing studies that have reported improvements in VO_2peak_ after vigorous or high intensity exercise interventions (Harrison et al., 2012; Roessler et al., 2013; Almenning et al., 2015; Costa et al., 2018) and highlights the importance of exercise intensity when prescribing exercise training in clinical practice or as part of a clinical trial.

A large proportion of women with PCOS are overweight or obese, with a recent meta-analysis reporting a pooled prevalence of 61% (Lim et al., 2012). It is therefore not surprising that many exercise and dietary interventions have an ultimate aim of reducing body weight/BMI. Modest weight loss of 5–10% in overweight women with PCOS is encouraged to yield clinical improvements (Teede et al., 2018a). However, it is important to note that health benefits can occur without significant weight loss (Hutchison et al., 2011; Sprung et al., 2013b; Covington et al., 2016). The lack of improvement in BMI following an exercise only intervention observed from our analysis is not surprising. However, when exercise is complimented with a dietary intervention, small, but clear, decreases in BMI can be achieved. In addition, our results support the inclusion of diet in order to promote improvements in WC. Research conducted by Thomson et al. (2008) reported reductions in body weight and waist circumference of ~10% across three different treatments groups (diet alone, diet + aerobic exercise, or diet + combined aerobic and resistance exercise) after 20 weeks. A study conducted by Bruner et al. (2006) reported no significant differences in body weight or BMI following an intervention of either nutritional counselling or a combined resistance training, aerobic exercise and nutritional counselling intervention. They did, however, report significant decreases in waist circumference of 5% in both groups following the intervention period. BMI as a measure of obesity is considered to have its limitations, with changes in BMI not necessarily reflecting changes in body fat (Rothman, 2008). Body composition assessment using direct methods such as dual-energy X-ray absorptiometry (DXA) may provide valuable information on changes in body composition. When deprived of DXA information, measures of WC may provide a better measure of obesity-related health risk than BMI (Janssen et al., 2004). It is possible that exercise training alone may have a limited impact on BMI but positively improves waist circumference or other markers of body composition, including increased lean mass and decreased fat mass, which can occur without changes in total body weight.

Insulin resistance is a key aetiological feature in PCOS and underpins the metabolic dysfunction present in women with PCOS (Dunaif et al., 1989; Diamanti-Kandarakis and Papavassiliou, 2006). Although not currently included in the diagnostic criteria, insulin resistance determined from insulin clamps is prevalent in 56–95% of women with PCOS (Stepto et al., 2013; Tosi et al., 2017; Li et al., 2019). It is therefore important to understand the impact of exercise type and intensity and its interaction with diet to explore effective exercise interventions to alleviate insulin resistance in women with PCOS before major complications occur. Resistance training is an effective treatment for improving insulin sensitivity in individuals with diabetes (Ishii et al., 1998; Holten et al., 2004; Ibañez et al., 2005), however, there is limited evidence to support the benefits of such training in PCOS (Thomson et al., 2008). We identified moderate decreases in HOMA-IR after resistance training interventions when compared to a control group. Resistance training is yet to be implemented in the treatment of PCOS, with current knowledge limited to few studies with small numbers of participants. However, there is evidence to support the effects of resistance training for improving insulin sensitivity in diabetic populations and therefore this may be applicable to women with PCOS. Our meta-analysis showed that vigorous intensity aerobic training also resulted in moderate decreases in HOMA-IR in women with PCOS. This is in line with findings from a number of other clinical populations (Pattyn et al., 2014; Weston et al., 2014; Jelleyman et al., 2015; Cassidy et al., 2017). Results from a study conducted by Greenwood et al. (2016) support the superior health benefits of vigorous exercise compared to moderate exercise in women with PCOS. They reported that 60 min of vigorous intensity exercise per week was associated with a 22% reduced odds of metabolic syndrome. In addition, Harrison et al. (2012) reported a 16% improvement in insulin sensitivity in women with PCOS following a 12-weeks vigorous intensity exercise intervention, as determined by the gold-standard euglycaemic-hyperinsulinaemic clamp method (DeFronzo et al., 1979). The use of the clamp method in clinical practice is impractical, however one must be cognisant that using HOMA-IR as a surrogate marker for IR has significant limitations which includes a low sensitivity in identifying IR (Tosi et al., 2017). Despite the pitfalls of using HOMA-IR to measure insulin resistance, most clinical research in PCOS continues to use this method due to its cost-effectiveness and ease of translation into clinical practice.

Elevated FAI is the most consistently observed androgenic abnormality in PCOS (Teede et al., 2011). Current research that measures FAI prior to and following an exercise intervention show contradictory results (Giallauria et al., 2008; Thomson et al., 2008; Stener-Victorin et al., 2012). This may relate to the complex relationship between FAI and insulin resistance, as the latter has profound effects on SHBG. Results from our meta-analysis could not provide any conclusive evidence in support of any type of exercise training or exercise intensity influencing FAI and is consistent with another recent meta-analysis (Kite et al., 2019). Our results suggest that resistance training may be the most likely to induce positive changes in FAI, however, due to the limited number of studies utilising resistance training, more research is required to validate this outcome. One study of 16 week study of progressive resistance training (*n* = 45) reported decreases in FAI values of 0.82% (Miranda-Furtado et al., 2016). In addition, a study comparing a 10-weeks intervention of either resistance training (*n* = 8) or high intensity interval training (*n* = 8) to a control group (*n* = 9), reported the largest decrease in FAI in the strength training group, with a decrease of −0.7% from baseline values (Almenning et al., 2015). Although resistance training shows promising results, reductions in FAI have also been reported after aerobic exercise (Randeva et al., 2002; Giallauria et al., 2008; Covington et al., 2015). Further research is required to determine the effective modality, dose and intensity of exercise for improvements in hyperandrogenism. There is also a need to identify more valid measures of androgen levels in women with PCOS to monitor impacts all interventions (e.g., exercise and/or diet, pharmacotherapies).

### Strengths and Limitations

An important strength of our analysis is the inclusion of a variety of study designs with well-characterised participants. This allowed us to go beyond existing systematic reviews and meta-analyses to generate a large dataset that included a no-intervention control group. We were also able to explore the modifying effects of diet, exercise intensity, training dose, and baseline values of the outcome measures, according to a particular current health and fitness level, enabling more individualised exercise prescription for women with PCOS. However, the inclusion of studies other than RCTs may be viewed as a limitation due to the possible increase in the risk of bias. However, all studies were assessed for bias and deemed of acceptable quality. It could also be argued that including a no-exercise control group in a study design could be considered of no additional use (Jones and Podolsky, 2015; Frieden, 2017) and it is established that in many clinical conditions, most outcomes impacted by exercise remain unchanged or worsen over the course of an intervention in no-exercise controls (Jelleyman et al., 2015). A limitation of this analysis is the large heterogeneity among the included studies with interventions varying greatly in frequency, intensity, and the extent of exercise supervision. Some studies had sparse description of the exercise interventions, further limiting our analysis. The inclusion of unsupervised exercise interventions may have under-estimated the benefits of exercise and future research should aim to document level of supervision to better gauge its effect on clinical outcomes.

## Conclusions

This work considerably expands on previous evidence and advances the knowledge of benefits of exercise prescription in women with PCOS. Our analysis demonstrates that exercise training in women with PCOS improves cardio-metabolic outcomes, both in the presence and independent of anthropometric changes, supporting the role of exercise therapy, as the first-line approach for improving health outcomes in women with PCOS. Specifically, for greater health improvements, exercise interventions and/or exercise prescription should aim to achieve and sustain a minimum of 20 h of vigorous intensity exercise over 10–12 weeks, equating to 120 min per week across this timeframe. Once achieving this goal, women should sustain this level of exercise for continued health maintenance. Resistance training also appears to have some health benefits and could be considered for women with PCOS. Adequate reporting of exercise intervention characteristics (i.e., exercise session supervision, exercise intensity, adherence, and compliance), use of gold-standard clinical outcome measures and consideration of long-term intervention sustainability is required through the application of high-quality, large clinical studies of longer duration required to provide definitive exercise prescription recommendations in women with PCOS.

## Data Availability Statement

Publicly available datasets were analyzed in this study. This data can be found here: Supplementary Table 3.

## Author Contributions

RP and RB conducted the literature search and data extraction. RP, RB, WH, and NS contributed to data analysis. RP, RB, and NS designed the figures and tables. All authors contributed to study design, data interpretation, and to writing and reviewing of the manuscript.

## Conflict of Interest

NS (CI) and CH (AI) have received funding from the NHMRC. TM has received funding from The Liaison Committee for Education, Research, and Innovation in Central Norway. The remaining authors declare that the research was conducted in the absence of any commercial or financial relationships that could be construed as a potential conflict of interest.
