# Supplementary material for: Exercise Interventions in Polycystic Ovary Syndrome: A Systematic Review and Meta-Analysis
Source: Front Physiol. 2020 Jul 7;11:606. doi: 10.3389/fphys.2020.00606 (PMC7358428; doi:10.3389/fphys.2020.00606)
Supplement: Supplementary file 1 [file Table_1.docx]

**Supplementary Table 1.** Search terms for systematic review

| **PCOS** | **Exercise** | **Limits** |
| --- | --- | --- |
| Polycystic ovary syndrome  or  Polycystic ovar*  or  PCO*  or  Stein Leventhal  or  Leventhal | And –  Exercise  or  Physical  or  Sport  or  Strength  or  Resistance  or  Lifestyle | Not –  Insulin Resistance |
