# Supplementary material for: Exercise Interventions in Polycystic Ovary Syndrome: A Systematic Review and Meta-Analysis
Source: Front Physiol. 2020 Jul 7;11:606. doi: 10.3389/fphys.2020.00606 (PMC7358428; doi:10.3389/fphys.2020.00606)
Supplement: Supplementary file 2 [file Table_2.docx]

**Supplementary Table 2.** Modified Downs and Black checklist for assessing methodological quality (Downs and Black, 1998).

| **Number** | **Question** | **Score** | | |
| --- | --- | --- | --- | --- |
| **Reporting** | | | | |
| 1 | Is the hypothesis/aim/objective of the study clearly described? | Yes = 1 | No = 0 |  |
| 2 | Are the main outcomes to be measured clearly described in Intro or Methods section? | Yes = 1 | No = 0 |  |
| 3 | Are the characteristics of the patients included in the study clearly described? | Yes = 1 | No = 0 |  |
| 4 | Are the interventions of interest clearly described? | Yes = 1 | No = 0 |  |
| 5 | Are the main findings of the study clearly described? | Yes = 1 | No = 0 |  |
| 6 | Does the study provide estimates of the random variability in the data for the main outcomes? (distribution - SE, SD, CI) | Yes = 1 | No = 0 |  |
| 7 | Did they report adherence to intervention? | Yes = 1 | No = 0 |  |
| 8 | Have all important adverse events that may be a consequence of the intervention been reported? | Yes = 1 | No = 0 |  |
| 9 | Have the characteristics of patients lost to follow-up been described? | Yes = 1 | No = 0 |  |
| 10 | Was all exercise supervised? | Yes = 1 | No = 0 | Unable to determine = 0 |
| 11 | Have actual probability values been reported (e.g. 0.035 rather than <0.05) for the main outcomes except where the p<0.001? | Yes = 1 | No = 0 | Unable to determine = 0 |
| **Validity - bias** | | | | |
| 12 | Was an attempt made to blind those measuring the main outcomes of the intervention? | Yes = 1 | No = 0 | Unable to determine = 0 |
| 13 | Were the statistical tests used to assess the main outcomes appropriate | Yes = 1 | No = 0 | Unable to determine = 0 |
| 14 | Was compliance with the intervention/s reliable? | Yes = 1 | No = 0 | Unable to determine = 0 |
| 15 | Were the main outcomes measures used accurate (valid and reliable) | Yes = 1 | No = 0 | Unable to determine = 0 |
| 16 | Were the patients in different intervention groups and control group recruited from the same population? | Yes = 1 | No = 0 | Unable to determine = 0 |
| 17 | Were study subjects in different intervention groups and control group recruited over the same period of time? | Yes = 1 | No = 0 | Unable to determine = 0 |
| 18 | Were study subjects randomised to intervention groups? | Yes = 1 | No = 0 | Unable to determine = 0 |
| 19 | Was the randomisation concealed from both patients and health care staff until baseline testing was complete? | Yes = 1 | No = 0 | Unable to determine = 0 |
| 20 | Were losses of patients to follow-up taken into account? | Yes = 1 | No = 0 | Unable to determine = 0 |
| **Power** | | | | |
| 21 | Did the study have sufficient power to detect a clinically important effect where the probability value for a difference being due to chance is less than 5% (size of smallest intervention group) | Yes = 1 | No = 0 | Unable to determine = 0 |
