# Supplementary material for: Exercise Interventions in Polycystic Ovary Syndrome: A Systematic Review and Meta-Analysis
Source: Front Physiol. 2020 Jul 7;11:606. doi: 10.3389/fphys.2020.00606 (PMC7358428; doi:10.3389/fphys.2020.00606)
Supplement: Supplementary file 3 [file Table_3.docx]

**Supplementary Table 3.** Summary of results from individual studies for each meta-analysed outcome measure.

|  |  |  |  | **VO_2peak_ (mL.kg.min^-1)^** | | | | | **BMI (kg/m^2^)** | | | | | **Waist Circumference (cm)** | | | | |
| --- | --- | --- | --- | --- | --- | --- | --- | --- | --- | --- | --- | --- | --- | --- | --- | --- | --- | --- |
| **Study** | **Diet** | **Group** | **N** | **Mean Baseline** | **Baseline SD** | **Delta** | **SE** | **SEM** | **Mean Baseline** | **Baseline SD** | **Delta** | **SE** | **SEM** | **Mean Baseline** | **Baseline SD** | **Delta** | **SE** | **SEM** |
| Almenning et al. [25] | No | RT | 8 | 39.3 | 10.2 | 0.9 | 0.9 | 1.7 | 27.1 | 6.6 | 0.4 | 0.3 | 0.6 | 93.7 | 17.6 | -1.4 | 0.7 | 1.4 |
|  | No | HIIT | 8 | 37.4 | 4.7 | 3.7 | 0.5 | 0.9 | 23.8 | 4.8 | 0.1 | 0.2 | 0.3 | 86.8 | 12.9 | 0.4 | 2.1 | 4.1 |
|  | No | Control | 9 | 36.8 | 7.8 | -0.8 | 0.8 | 1.7 | 26.3 | 5.2 | -0.2 | 0.3 | 0.7 | 92.6 | 15.5 | -0.3 | 1.6 | 3.4 |
| Bruner et al. [26] | Yes | AE | 7 |  |  |  |  |  | 36.2 | 5.3 | -0.3 | 0.8 | 1.4 | 98.3 | 13.2 | -5.2 | 0.9 | 1.6 |
|  | Yes | Control | 5 |  |  |  |  |  | 37.1 | 7.6 | -1.2 | 0.9 | 1.5 | 99.8 | 11.2 | -5 | 0.6 | 0.9 |
| Costa et al. [27] | No | AE | 14 | 27.9 | 3.3 | 5.9 | 0.9 | 2.3 | 32.0 | 4.2 | -0.7 | 0.2 | 0.6 | 92.8 | 10.3 | -3.7 | 1.3 | 3.3 |
|  | No | Control | 13 | 26.8 | 6.2 | 0.2 | 0.9 | 2.3 | 33.6 | 5.1 | 0.7 | 0.4 | 0.9 | 94.1 | 11.6 | 3.8 | 1.3 | 3.2 |
| Curi et al. [39] | Yes | AE + RT | 12 |  |  |  |  |  | 31.8 | 1.6 | -1.7 | 0.5 | 1.2 | 101.8 | 3.9 | -6.7 | 1.5 | 3.7 |
| Giallauria et al. [35] | No | AE | 62 | 19.0 | 3.9 | 5.5 | 1.6 | 8.9 | 29.2 | 2.9 | -1.3 | 0.1 | 0.7 |  |  |  |  |  |
|  | No | Control | 62 | 18.8 | 3.3 | -0.2 | 0.2 | 0.9 | 29.5 | 3.5 | -0.2 | 0.1 | 0.7 |  |  |  |  |  |
| Hutchinson et al. [44], Moran et al.[45], Hutchinson et al. [36], Harrison et al. [46] | No | AE | 13 | 25.9 | 6.5 | 5.5 | 1.8 | 4.6 | 35.6 | 5.8 | -0.6 | 0.3 | 0.8 | 104.0 | 13.3 | -1.0 | 1.2 | 3.0 |
| Ladson et al. [29] | Yes | AE | 16 |  |  |  |  |  |  |  |  |  |  | 107.1 | 18.4 | -4.1 | 1.1 | 3.2 |
| Miranda-Furtado et al. [37]  Kogure et al. [47]  Kogure et al. [48] | No | RT | 45 |  |  |  |  |  | 28.5 | 6.0 | -0.2 | 0.1 | 0.7 | 81.7 | 12.8 | -1.2 | 0.3 | 1.6 |
| Moro et al. [49]  Redman et al. [50]  Covington et al. [51]  Covington et al. [34] | No | AE | 8 | 27.5 | 1.3 | 3.4 | 1.0 | 1.9 | 32.1 | 5.2 | -0.3 | 0.6 | 1.2 |  |  |  |  |  |
| Nybacka et al. [30]  Nybacka et al. [52] | No | AE | 17 |  |  |  |  |  | 34.9 | 5.3 | -0.9 | 0.4 | 1.2 |  |  |  |  |  |
|  | Yes | AE | 12 |  |  |  |  |  | 38.8 | 7.9 | -1.9 | 0.9 | 2.1 |  |  |  |  |  |
|  | Yes | Control | 14 |  |  |  |  |  | 34.7 | 5 | -1.7 | 0.4 | 1.1 |  |  |  |  |  |
| Orio et al. [31] | Yes | AE | 39 | 19.0 | 2.1 | 6 | 1.8 | 7.8 | 26.7 | 2.8 | -1.4 | 0.1 | 0.6 |  |  |  |  |  |
|  | No | Control | 50 | 19.0 | 2.7 | -0.5 | 0.3 | 1.6 | 27.0 | 2.9 | -0.1 | 0.1 | 0.6 |  |  |  |  |  |
| Orio et al. [40] | Yes | AE | 32 | 17.5 | 2.2 | 8.1 | 2.2 | 8.9 | 28.9 | 3 | -2.1 | 0.2 | 0.7 | 94.6 | 4.3 | -5.1 | 1.4 | 5.6 |
|  | Yes | AE | 32 | 17.2 | 2.3 | 5.9 | 1.6 | 6.5 | 28.9 | 2.3 | -1.2 | 0.2 | 0.7 | 95.1 | 4.4 | -3 | 0.8 | 3.3 |
| Randeva et al. [38] | No | AE | 12 |  |  |  |  |  | 34.0 | 4.5 | -0.2 | 0.2 | 0.7 |  |  |  |  |  |
|  | No | Control | 9 |  |  |  |  |  | 37.6 | 9.7 | 0.7 | 0.4 | 0.9 |  |  |  |  |  |
| Roessler et al. [41] | No | AE | 8 | 25.0 |  | 3.2 | 1.5 | 2.9 | 34.8 | 7.1 | -0.4 | 0.2 | 0.4 | 112.2 | 14.1 | -6.0 | 2.5 | 5.15.1 |
| Sprung et al. [42] | No | AE | 6 | 27.1 | 5.2 | 5.0 | 1.6 | 2.8 | 31.0 | 5.7 | -0.3 | 0.5 | 0.8 | 100.0 | 15.7 | -4.0 | 2.6 | 4.5 |
| Sprung et al. [43] | No | AE | 10 | 29.0 | 5.4 | 4.7 | 0.8 | 1.8 | 31.0 | 4.2 | -0.2 | 0.3 | 0.7 | 100.0 | 7.7 | -3.0 | 2.0 | 4.5 |
|  | No | Control | 7 | 23.8 | 2.5 | -0.5 | 1.1 | 2.1 | 35.0 | 4.9 | 0.2 | 0.2 | 0.4 | 109.0 | 15.7 | -1.0 | 1.5 | 2.8 |
| Stener-Victorin et al. [53, 54] &  Jedel et al. [28] | No | AE | 30 | 33.9 | 8.5 | 4.1 | 1.4 | 5.5 | 27.7 | 6.4 | 0.1 | 0.2 | 0.8 | 91.1 | 13.9 | -0.6 | 0.4 | 1.6 |
|  | No | Control | 15 | 35.2 | 9.3 | 2.2 | 0.6 | 1.6 | 26.8 | 5.6 | 0.2 | 0.2 | 0.5 | 86.0 | 12.0 | -0.1 | 0.3 | 0.9 |
| Thomson et al. [32, 55, 56] | Yes | AE | 18 | 25.5 | 3.7 | 4.4 | 1.0 | 3.1 |  |  |  |  |  | 100.2 | 12.2 | -11.7 | 1.4 | 4.3 |
|  | Yes | AE + RT | 20 | 24.4 | 2.8 | 2.6 | 0.6 | 1.9 |  |  |  |  |  | 103.8 | 12.6 | -11.0 | 1.4 | 4.5 |
|  | Yes | Control | 14 | 23.8 | 3.7 | -0.5 | 0.8 | 2.1 |  |  |  |  |  | 103.0 | 12.6 | -10.8 | 1.9 | 5.0 |
| Vigorito et al. [33] | No | AE | 45 | 17.6 | 2.5 | 6.1 | 1.7 | 8.2 | 29.3 | 2.9 | -1.3 | 0.1 | 0.7 | 94.5 | 3.4 | -2.7 | 0.8 | 3.6 |
|  | No | Control | 45 | 17.7 | 2.2 | 0.2 | 0.2 | 1.1 | 29.4 | 3.5 | -0.1 | 0.1 | 0.7 | 94.0 | 3.0 | -0.2 | 1.2 | 5.8 |

Supplementary Table 3 continued…

|  | **HOMA-IR** | | | | | **FAI** | | | | |
| --- | --- | --- | --- | --- | --- | --- | --- | --- | --- | --- |
| **Study** | **Mean Baseline** | **Baseline SD** | **Delta** | **SE** | **SEM** | **Mean Baseline** | **Baseline SD** | **Delta** | **SE** | **SEM** |
| Almenning et al. [25] | 3.3 | 1.3 | -0.3 | 0.3 | 0.6 | 2.8 | 1.7 | -0.7 | 0.3 | 0.6 |
|  | 4.9 | 1.7 | -0.8 | 0.3 | 0.6 | 1.5 | 1.2 | 0.4 | 0.9 |  |
|  | 3.6 | 2.1 | 0.7 | 0.5 | 1.0 | 2.6 | 1.4 | 0 | 0.5 | 1.1 |
| Bruner et al. [26] |  |  |  |  |  | 13.9 | 6.9 | 0 | 1.8 | 3.6 |
|  |  |  |  |  |  | 20.3 | 10.3 | -0.5 | 3.6 | 5.2 |
| Costa et al. [27] | 3.0 | 2.0 | -0.6 | 0.7 | 1.9 |  |  |  |  |  |
|  | 1.5 | 1.0 | -0.5 | 0.3 | 0.8 |  |  |  |  |  |
| Curi et al. [39] | 3.4 | 0.8 | -0.6 | 0.8 | 1.9 |  |  |  |  |  |
| Giallauria et al. [35] |  |  |  |  |  | 10.1 | 7.9 | -1.8 | 1.3 | 9.8 |
|  |  |  |  |  |  | 9.7 | 8.1 | 0.1 | 1.4 | 11.1 |
| Hutchinson et al. [44], Moran et al.[45], Hutchinson et al. [36], Harrison et al. [46] |  |  |  |  |  | 10.7 | 5.0 | -0.6 | 1.0 | 2.8 |
| Ladson et al. [29] |  |  |  |  |  | 12.5 | 8.8 | -1.6 | 1.6 | 5.1 |
| Miranda-Furtado et al. [37]  Kogure et al. [47]  Kogure et al. [48] | 2.3 | 1.9 | 0.2 | 0.3 | 1.2 | 8.3 | 6.4 | -0.8 | 0.3 | 1.8 |
| Moro et al. [49]  Redman et al. [50]  Covington et al. [51]  Covington et al. [34] | 3.6 | 2.7 | -0.1 | 0.5 | 1.0 | 18.8 | 14.2 | -1.6 | 1.3 | 2.8 |
| Nybacka et al. [30]  Nybacka et al. [52] |  |  |  |  |  |  |  |  |  |  |
|  |  |  |  |  |  |  |  |  |  |  |
|  |  |  |  |  |  |  |  |  |  |  |
| Orio et al. [31] | 4.2 | 1.2 | -1.0 | 0.3 | 1.2 | 13.3 | 6.4 | -0.3 | 1.5 | 7.9 |
|  | 4.0 | 1.1 | 0.2 | 0.3 | 1.3 | 13.0 | 5.9 | -0.2 | 1.3 | 7.7 |
| Orio et al. [40] |  |  |  |  |  | 8.5 | 2.1 | 0.3 | 1.1 | 5.1 |
|  |  |  |  |  |  | 8.5 | 2.4 | 0.1 | 1.1 | 5.1 |
| Randeva et al. [38] |  |  |  |  |  | 9.0 | 5.7 | -0.9 | 0.9 | 2.3 |
|  |  |  |  |  |  | 12.7 | 8.8 | -2.1 | 1.5 | 3.3 |
| Roessler et al. [41] |  |  |  |  |  |  |  |  |  |  |
| Sprung et al. [42] | 3.5 | 2.3 | 0.5 | 1.1 | 1.8 | 8.5 | 2.5 | 0.7 | 0.8 | 1.4 |
| Sprung et al. [43] | 3.4 | 3.2 | 0.4 | 0.3 | 0.7 | 8.7 | 9.8 | 1.1 | 0.6 | 1.4 |
|  | 3.4 | 1.9 | 1.1 | 0.5 | 1.0 | 8.7 | 9.3 | 0.6 | 0.7 | 1.4 |
| Stener-Victorin et al. [53, 54] &  Jedel et al. [28] | 1.6 | 1.0 | -0.3 | 0.8 | 1.2 | 7.1 | 4.3 | 1.7 | 1.1 | 1.8 |
|  | 2.0 | 1.5 | -0.4 | 0.3 | 0.6 | 5.8 | 4.0 | -0.4 | 0.8 | 1.5 |
| Thomson et al. [32, 55, 56] | 1.9 | 1.0 | -0.5 | 0.2 | 0.6 | 8.5 | 5.6 | -2.6 | 1.0 | 3.3 |
|  | 2.0 | 1.0 | -0.6 | 0.2 | 0.6 | 9.1 | 5.7 | -2.9 | 0.9 | 3.1 |
|  | 2.3 | 1.0 | -0.6 | 0.2 | 0.6 | 11.2 | 5.5 | -2.8 | 1.1 | 3.3 |
| Vigorito et al. [33] |  |  |  |  |  | 8.5 | 3.4 | 0.2 | 1.3 | 8.2 |
|  |  |  |  |  |  | 8.6 | 3.6 | -0.1 | 1.3 | 8.3 |

VO_2peak_ – Peak Oxygen Consumption, BMI – Body Mass Index, HOMA-IR – Homeostatic Model Assessment of Insulin Resistance, FAI – Free Androgen Index, SE – Standard Error, SEM – Standard Error of Measurement, RT – Resistance Training, HIIT – High Intensity Interval Training, AE – Aerobic Exercise
