# Supplementary material for: Exercise Interventions in Polycystic Ovary Syndrome: A Systematic Review and Meta-Analysis
Source: Front Physiol. 2020 Jul 7;11:606. doi: 10.3389/fphys.2020.00606 (PMC7358428; doi:10.3389/fphys.2020.00606)
Supplement: Supplementary file 4 [file Table_4.docx]

**Supplementary Table 4.** Magnitude thresholds based on standardisation factors.

|  | **Beneficial effects (%)** | | | | |
| --- | --- | --- | --- | --- | --- |
|  | **Small** | **Mod** | **Large** | **V.Large** | **X.Large** |
| **VO_2peak_** | 5.0 | 16 | 34 | 63 | 170 |
| **BMI** | -4.0 | -12 | -22 | -33 | -56 |
| **WC** | -2.5 | -7.3 | 14 | -22 | -40 |
| **FAI** | -12 | -32 | -54 | -72 | -92 |
| **HOMA-IR** | -12 | -32 | -54 | -72 | -92 |
|  | **Harmful effects (%)** | | | | |
| **VO_2peak_** | -5.0 | -14 | -27 | -40 | -64 |
| **BMI** | 4.0 | 13 | 27 | 48 | 120 |
| **WC** | 2.5 | 7.7 | 16 | 28 | 64 |
| **FAI** | 12 | 41 | 97 | 210 | 860 |
| **HOMA-IR** | 12 | 41 | 97 | 210 | 860 |

Mod – Moderate, V.Large – Very Large, X-Large – Extra Large, VO_2peak_ – Peak Oxygen Consumption, BMI – Body Mass Index, WC – Waist Circumference, FAI – Free Androgen Index, HOMA-IR – Homeostatic Model Assessment of Insulin Resistance.
